# Postherpetic Pruritus: A Potential Complication of Herpes Zoster Virus Infection

**DOI:** 10.7759/cureus.5665

**Published:** 2019-09-16

**Authors:** Shahzeb Hassan, Philip R Cohen

**Affiliations:** 1 Dermatology, Northwestern University Feinberg School of Medicine, Chicago, USA; 2 Dermatology, San Diego Family Dermatology, San Diego, USA

**Keywords:** chicken, herpes, itch, pox, pruritus, varicella, zoster

## Abstract

Postherpetic pruritus is an uncommon adverse sequela of Varicella zoster infection. It can present with or without prior postherpetic neuralgia. A 57-year-old woman who developed persistent postherpetic pruritus following a Varicella zoster infection, affecting the skin between her right thoracic fifth to eighth dermatomes is described; she did not have postherpetic neuralgia. Her condition did not improve with systemic antiviral or gabapentin treatment; however, nine years later, she exhibited significant relief after two months of acyclovir 800 mg five times daily. In summary, postherpetic pruritus is a potential complication that can occur alone or in combination with postherpetic neuralgia. Some patients with postherpetic pruritus have a treatment-refractory disease. However, other patients respond to gabapentin; yet, long-term interventions may be necessary for persistent pruritus. Our patient’s pruritus significantly improved after restarting systemic antiviral therapy.

## Introduction

Herpes zoster can result in temporary or permanent postherpetic neuralgia [1]. Postherpetic pruritus is another adverse event that can occur alone or in combination with postherpetic neuralgia [2]. A woman with postherpetic pruritus, who does not have postherpetic neuralgia, is described and the features of this uncommon condition are reviewed.

## Case presentation

A 57-year-old African American woman presented with itching on her right side in 2019. The itching began in 2010 following an episode of varicella zoster virus infection that affected the skin between her fifth to eighth right thoracic dermatomes. Her Herpes zoster infection was treated with acyclovir (800 mg, five times a day) for 10 days. She did not experience any pain or postherpetic neuralgia. However, shortly after the blisters resolved, the affected area became pruritic; in addition, she also noted that exposure to cold temperature made the itch worse.

Her past medical history is significant for cervical cancer, diabetes mellitus (type 2), scleroderma, and systemic lupus erythematosus. Previously, she also had culture-confirmed Varicella zoster virus infections not only in 1999 affecting her seventh to eighth right cervical dermatomes (right hand and fourth finger) but also in 2002 affecting her sixth to seventh right cervical dermatomes (right arm). She has arthritic joint pains and is currently receiving gabapentin, hydroxychloroquine, and prednisone each day.

**Figure 1:**
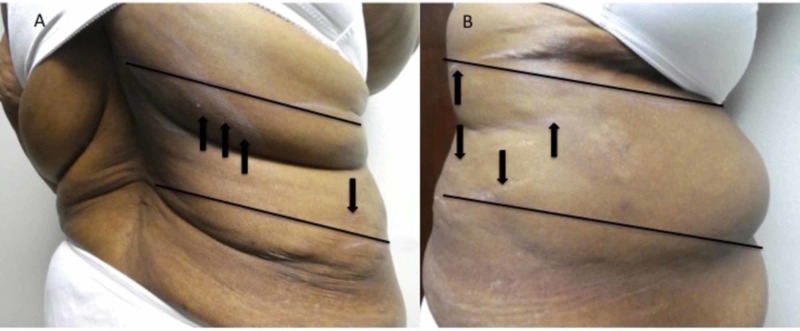
Postherpetic pruritus extending from the middle of the back to the abdomen on the right side of a 57-year-old woman Pruritus is experienced in a region of skin involving the back (A), flank (A and B), and abdomen (B) from the right fifth to eighth thoracic dermatomes (between the black lines). Numerous excoriations (black arrows) result from her scratching the pruritic area.

Cutaneous examination was performed. She had pruritus originating on her right mid-back that extended to involve her right flank and right abdomen. The distribution of her pruritus involved the skin between her right fifth to eighth thoracic dermatomes; excoriations were also present in the affected area on her right side (Figure 1).

**Figure 2:**
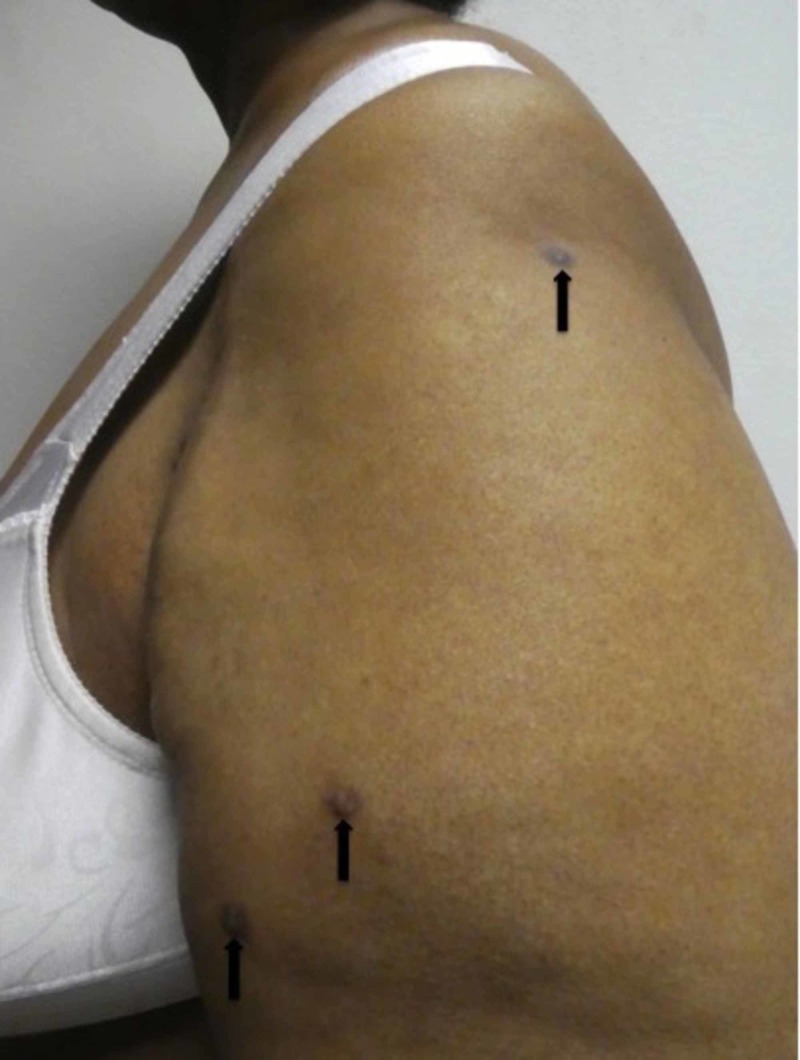
Dermatofibromas on the left arm of 57-year-old woman with postherpetic pruritus Dermatofibromas, presenting as three hyperpigmented dermal nodules (black arrows), are present on the left arm of a woman with systemic lupus erythematous who is being treated with prednisone.

She also had nine hyperpigmented dermal nodules, ranging in size from five to eight millimeters in diameter. They were located on her left arm (three nodules; Figure 2), left abdomen (two nodules), left buttock, left dorsal foot, left hip and right lower back. When the skin adjacent to the nodule was squeezed between the index finger and the thumb, a positive dimple sign - demonstrated by depression of the dermal nodule - would occur.

Correlation of her medical history and clinical presentation established the diagnosis of postherpetic pruritus. She also had multiple dermatofibromas on her body and extremities.

She was already receiving gabapentin (600 mg, three times a day), hydroxychloroquine (200 mg, twice daily), and prednisone (2.5 mg, once daily). Hydroxyzine (25 mg, three times a day) initially provided temporary relief of her pruritus. Acyclovir (800 mg, five times a day) was also started. There was a significant improvement of her pruritus at her follow up visit eight weeks later. She continued antiviral therapy.

## Discussion

Herpes zoster is caused by reactivation of the Varicella zoster virus in sensory ganglia. Varicella zoster infection has several potential complications including secondary bacterial infections and postherpetic neuralgia. In addition, some patients develop postherpetic pruritus (which has also been referred to as postherpetic itch) [3].

Postherpetic pruritus is characterized by chronic and persistent itching in dermatomes previously affected by herpes zoster. A study of 100 patients with postherpetic neuralgia found that 53 of these patients also experienced postherpetic pruritus. The development of postherpetic pruritus was more frequent amongst the individuals who experienced facial herpes zoster infection [2]. Although postherpetic pruritus most commonly occurred in patients who also had postherpetic neuralgia, some of the affected individuals did not have prior or concurrent postherpetic neuralgia; however, the incidence of postherpetic pruritus in the absence of postherpetic neuralgia remains to be determined [4-6].

The pathogenesis of postherpetic pruritus remains to be established. Varicella zoster infection may have an effect on central itch-specific neurons [4]. Nonetheless, the co-occurrence of pain and itching is thought to involve reduced subepidermal nerve fiber density in postherpetic neuralgia [7]. 

It may be postulated that the location of postherpetic pruritus is related to a site of immune dysregulation caused by the previous skin damage. Essentially, an immunocompromised cutaneous district is created by the Varicella zoster infection. Subsequently, the pruritus develops in the dermatomal distribution of the prior viral infection [8-11].

The onset of postherpetic pruritus is variable. It can occur immediately following the resolution of blisters. Alternatively, it can begin several weeks after the herpes zoster infection resolves [2].

The severity of postherpetic pruritus varies. Similar to our patient, excoriations - secondary to scratching in the affected dermatomes - may be present.

The treatment of postherpetic pruritus may be complex since neuropathic itch may not respond to antihistamines or other common medications for pruritus [4]. Gabapentin has been used successfully to treat postherpetic pruritus [6]. However, gabapentin does not resolve postherpetic pruritus in all individuals; our patient experienced no relief of the gabapentin that she was taking for her chronic arthritis.

Several other interventions have been used in an attempt to manage this condition (Table 1) [5-6,12-14]. Individual reports described patients whose postherpetic pruritus has been effectively treated with these modalities. Our patient’s pruritus was significantly improved after eight weeks of acyclovir. However, some of the therapies, such as those used for postherpetic neuralgia, can actually make the pruritus worse [2].

**Table 1:** Successful treatment options for postherpetic pruritus C, centigrade; CR, current report; d, day; °, degrees; mg, milligrams; mL, milliliters; %, percent; Ref, reference [5-6], [12-14] ^a^A 37-year-old man who developed lesions of postherpetic pruritus in the right arm and the upper trunk was unsuccessfully treated with steroid creams, antihistamines, sedatives, analgesics, and carbamazepine. This individual also had pain in his right upper arm prior to the onset of the pruritus. This case demonstrates the potential resistance to treatment that characterizes postherpetic pruritus and neuralgia [15].

Treatment^a^	Comment	Ref
Acyclovir	A 57-year-old woman improved with two months of acyclovir.	CR
Amitriptyline and ketamine gel	A 64-year-old man was initially treated with a variety of medications, including hydrocortisone cream (2.5%, twice daily), dilute acetic acid, gabapentin (400 mg, three times a day), topical lidocaine patches, and oral hydroxyzine (25 mg daily). These treatments only decreased the patient’s pruritus from 10/10 to 7-8/10 and the duration was less than 24 hours. Subsequent incorporation of topical 2% amitriptyline/0.5% ketamine gel resulted in a modest improvement of the pruritus.	Griffin JR et al. (2015)
Carbamazepine and hydroxyzine	A 22-year-old man was treated with hydroxyzine (75 mg/d) and carbamazepine (200 mg/d) for 3 days. The pruritus improved from 10/10 to 7/10. Subsequently, the dose of carbamazepine was increased to 400 mg/d while the dose of hydroxyzine dose was decreased to 50 mg/d (because of drowsiness). Two weeks of this treatment regimen completely resolved the patient’s pruritus.	Semionov V et al. (2008)
Gabapentin	A 40-year-old woman was started on gabapentin. Three weeks later, the patient reported no symptoms of pruritus.	Kroshinsky D et al. (2011)
Pulsed radiofrequency	A 56-year-old man underwent pulsed radiofrequency at the great occipital nerve (37°C for 120 seconds and 42°C for 120 seconds). He was also given diprospan (1 mg), vitamin B12 (0.5 mg), and lidocaine plain (1ml of 1%). His itching intensity improved from 5/10 to 1/10, but his pain worsened from 2/10 to 5/10. Pulsed radiofrequency was then performed at the great occipital nerve and supraorbital nerve. The patient was also given gabapentin (300 mg once a day). At one week follow-up, patient’s pain severity reduced to 1/10. The gabapentin dosage was lowered to 200 mg. At three weeks follow-up, the patient did not have symptoms and the patient’s gabapentin dosage was reduced further to 100 mg. At 12 weeks follow-up, the patient was asymptomatic.	Ding DF et al. (2014)
Serial stellate ganglion blocks with 0.25% bupivacaine	A 10-year-old man was given two serial stellate ganglion blocks; each dose was 0.5 mL of 0.25% bupivacaine. After recovery, the patient was noted to be free of any itching. Subsequent blocks were performed on days three and six following the initial procedure. At two weeks follow-up, notable improvements were seen in the wounds. The patient was maintained on a benzodiazepine and an antihistamine. Four months later, the patient still had pruritus, but it was noted to have improved considerably.	Peterson RC et al. (2009)

Clinicians should conscientiously evaluate pruritus in herpes zoster patients. Itching can have a significant impact on the physical and psychological well-being of these individuals. It can affect the ability of the patient to perform everyday tasks and interact effectively within their social environments [16].

The patient with postherpetic pruritus may continuously scratch the area, particularly if it is desensitized to pain, and create ulcers to the bone [4]. In addition, postherpetic pruritus can affect potential treatment regimens; for example, opioids may not be able to be incorporated into the management of patients with concurrent pruritus and pain following Varicella zoster virus infection. Additional retrospective and prospective studies are warranted to investigate the pathogenesis and potential therapeutic options for postherpetic pruritus.

The cause of our patient’s post-herpetic pruritus remains to be established. Perhaps her persistent pruritus was related to a low level of Varicella zoster virus activity due to her chronic immunosuppression. This possibility would account for why she benefited from repeat antiviral therapy.

Herpes zoster is a common viral infection amongst patients with systemic lupus erythematosus, but its exact connection with postherpetic itch is not known [17]. In addition, multiple dermatofibromas have been found in individuals with systemic lupus erythematous or receiving immunosuppressive therapy (such as prednisone) or both [18]. Our patient had systemic lupus erythematous; she also had recurrent episodes of Varicella zoster virus infection and multiple dermatofibromas.

## Conclusions

Postherpetic pruritus is an uncommon adverse sequela following Varicella zoster infection. It can occur as an isolated event or associated with postherpetic neuralgia. Some patients improve with treatments for postherpetic neuralgia. Indeed, our patient experienced significant improvement of her chronic and persistent postherpetic pruritus after receiving oral acyclovir - at a dosage typically used for acute Varicella zoster infection - for two months.
